# Automatic generation of subject-specific finite element models of the spine from magnetic resonance images

**DOI:** 10.3389/fbioe.2023.1244291

**Published:** 2023-09-05

**Authors:** Joeri Kok, Yulia M. Shcherbakova, Tom P. C. Schlösser, Peter R. Seevinck, Tijl A. van der Velden, René M. Castelein, Keita Ito, Bert van Rietbergen

**Affiliations:** ^1^ Department of Biomedical Engineering, Eindhoven University of Technology, Eindhoven, Netherlands; ^2^ Image Sciences Institute, University Medical Center Utrecht, Utrecht, Netherlands; ^3^ Department of Orthopaedic Surgery, University Medical Center Utrecht, Utrecht, Netherlands; ^4^ MRIguidance BV, Utrecht, Netherlands

**Keywords:** synthetic computed tomography, deep-learning, mesh morphing, personalized medicine, vertebra, intervertebral disc

## Abstract

The generation of subject-specific finite element models of the spine is generally a time-consuming process based on computed tomography (CT) images, where scanning exposes subjects to harmful radiation. In this study, a method is presented for the automatic generation of spine finite element models using images from a single magnetic resonance (MR) sequence. The thoracic and lumbar spine of eight adult volunteers was imaged using a 3D multi-echo-gradient-echo sagittal MR sequence. A deep-learning method was used to generate synthetic CT images from the MR images. A pre-trained deep-learning network was used for the automatic segmentation of vertebrae from the synthetic CT images. Another deep-learning network was trained for the automatic segmentation of intervertebral discs from the MR images. The automatic segmentations were validated against manual segmentations for two subjects, one with scoliosis, and another with a spine implant. A template mesh of the spine was registered to the segmentations in three steps using a Bayesian coherent point drift algorithm. First, rigid registration was applied on the complete spine. Second, non-rigid registration was used for the individual discs and vertebrae. Third, the complete spine was non-rigidly registered to the individually registered discs and vertebrae. Comparison of the automatic and manual segmentations led to dice-scores of 0.93–0.96 for all vertebrae and discs. The lowest dice-score was in the disc at the height of the implant where artifacts led to under-segmentation. The mean distance between the morphed meshes and the segmentations was below 1 mm. In conclusion, the presented method can be used to automatically generate accurate subject-specific spine models.

## 1 Introduction

Subject-specific finite element (FE) models of the spine are often used in research to evaluate the design of treatments (e.g., when designing cages for spinal fusion (Loenen et al., 2022) and braces for the correction of scoliosis (Vergari et al., 2015)), bone strength (Faulkner et al., 1991) and the progression of load-adaptive processes (Rijsbergen et al., 2018). The generation of such models is typically based on computed tomography (CT) scans from which the 3D geometry of the vertebrae as well as their density can be derived. However, such scans provide little information about intervertebral disc (IVD) shape and properties, and requires the exposure of subjects to harmful radiation, which in particular for children is a major issue (Miglioretti et al., 2013). Magnetic resonance (MR) imaging is better suited for the visualization of soft tissues such as the IVD and does not include ionizing radiation exposure but provides very limited information about osseous structures. Several studies therefore combined CT and MR images in order to obtain subject-specific information for both bone and IVD, but this approach obviously requires scanning subjects with both modalities, still involves harmful radiation, and requires co-registration of CT and MR data, which is not trivial (Castro-Mateos et al., 2016).

New techniques based on deep-learning are emerging that allow for the generation of synthetic CT (sCT) scans based on MR scans (Florkow et al., 2020; Parrella et al., 2023; Zhao et al., 2023). These synthetic scans resemble CT images but are based on a set of MR sequences only. Using deep-learning techniques, the image information obtained from the different sequences are translated to Hounsfield Units. Since these scans are inherently aligned with the original MR scans, soft tissues and hard tissues can be segmented and modelled without the requirement for co-registration of images or structures. However, due to the complexity of the spine geometry, accurate segmentation of these images and their translation to finite element models is challenging.

With increases in computational power and the development of U-Net deep-learning strategies (Ronneberger et al., 2015), automatic segmentation of anatomical structures has become more feasible. Many of the biomedical image analysis competitions in the last decade involved the development of an automatic segmentation algorithm (Maier-Hein et al., 2018). In these challenges datasets containing images and resulting deep-learning networks entered in the competition are often openly accessible. For the spine, the top performing deep-learning networks, based on the U-Net structure, were able to label vertebrae with an accuracy over 94% reaching a dice-score of 0.9 for the segmentation of vertebrae (Payer et al., 2020). Isensee et al. developed a self-configuring framework including a U-Net structure that can be used as an out-of-the-box tool for many biomedical image segmentation purposes (Isensee et al., 2021). It is expected that these networks can also be employed for the automatic segmentation of vertebrae and IVDs from MR and sCT images.

To further automate the generation of subject-specific FE models of the spine, meshing should also be performed automatically. Due to the complexity of the spine, including vertebrae, IVDs, and ligaments, creating a good quality mesh can be difficult. A commonly used approach is to use a template mesh including all desired structures that can then be morphed to a subject-specific target geometry using non-rigid registration. Since spine models are mostly based on CT scans, morphing is generally performed on individual vertebrae (Hadagali et al., 2018; Rubenstein et al., 2022), where then later the IVDs can be added through interpolation (Campbell and Petrella, 2015; Caprara et al., 2021). With the use of co-registered CT and MR, promising results have been shown when morphing a lumbar spine mesh to segmentations of the vertebrae and IVDs (Castro-Mateos et al., 2016). Non-rigid registration with (variations on) iterative closest point algorithms has long been regarded as the state-of-the-art. Recently, a Bayesian coherent point drift (BCPD) algorithm has been developed (Hirose, 2021a). This algorithm can be seen as an adaptation of a previously defined coherent point drift algorithm (Myronenko and Song, 2010)with the added benefits that it always converges and can easily be accelerated.

The aim of this paper is to develop a new approach to create patient-specific FE models of the spine in a fully automated manner from MR images and their derived sCT images. This approach combines special MR sequences, deep-learning algorithms for sCT generation and the automatic segmentation of the IVDs and vertebrae, and mesh morphing using a BCPD algorithm. A specific aim of this study was to test the accuracy of the generated meshes for normal spines as well as for a scoliotic case.

## 2 Methods

### 2.1 Image acquisition

**FIGURE 1:**
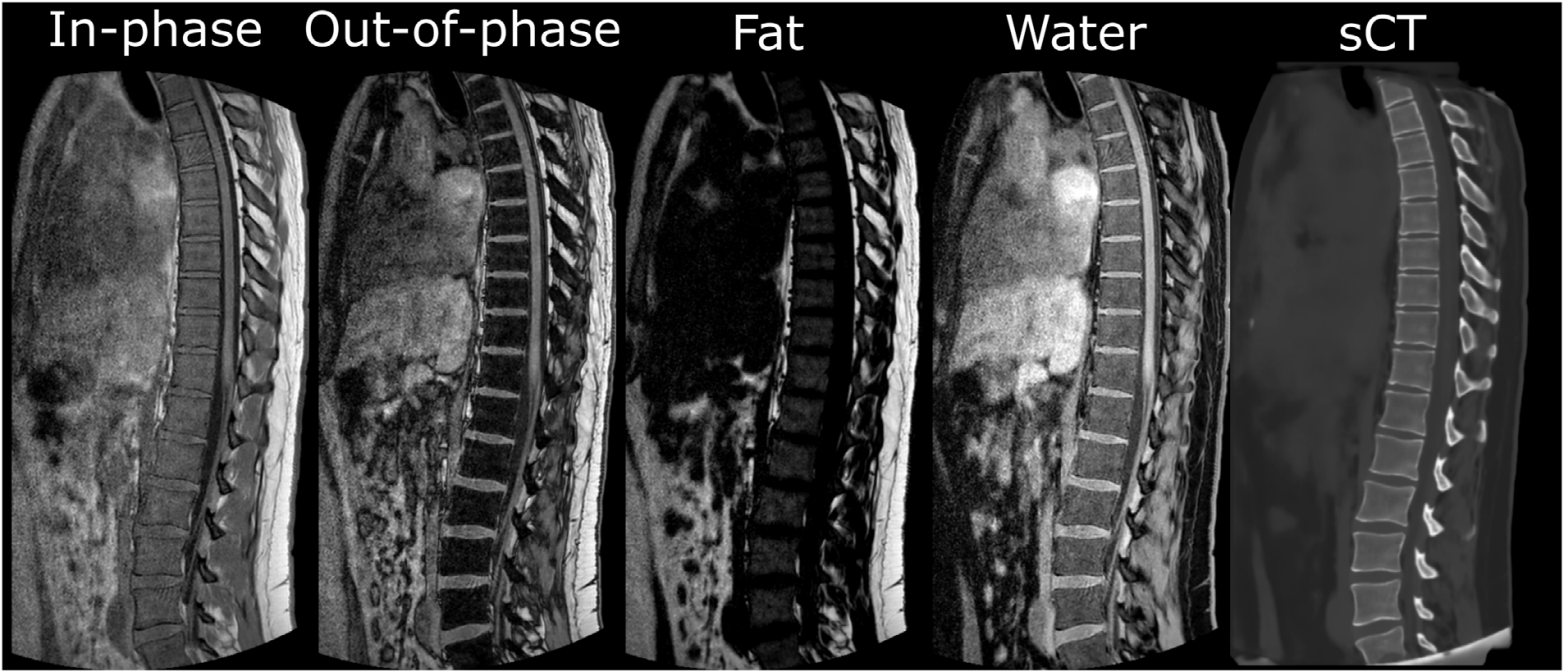
Sagittal slices from the 3D in- and out-of-phase images, the fat and water reconstructed images, and the generated synthetic CT images.

The study was eligible for expedited review as decided by the local METC (protocol ID 15–466). Eight adult volunteers were recruited and provided informed and written consent for MR imaging of the thoracic and lumbar spine (Table 1). Out of the eight volunteers, six have no known spine morbidities, one has scoliosis (∼30° Cobb angle), and one has undergone L4-S1 spinal fusion surgery (SUSTAIN-O interbody Spacer made of PEEK, REVERE Stabilization System for rods, screws and connectors, TRANSITION Stabilization System for the semi-rigid components; Globus Medical, Audubon, United States). 3D Sagittal in-phase, and out-of-phase scans were taken of the spine on a 1.5 tesla MR scanner (Philips Healthcare, Best, Netherlands, software release 5.7) using the built in posterior coil (Figure 1). RF-spoiled T1-weighted multi-echo-gradient-echo sagittal MR images were acquired with the following parameter settings: field of view (AP × FH × LR) 220 × 420 × 100 mm^3^, acquisition voxel size 1 × 1 × 2 mm^3^, reconstruction voxel size 0.625 × 0.625 × 1 mm^3^, flip angle 10°, TR/TE1/TE2 = 7 ms/2.1 ms/4.2 ms, and scan duration = 4 min 48 s. TE1 and TE2 were chosen for out-of-phase and in-phase, where water and fat images at 1.5 tesla were automatically reconstructed by the MR system using the Dixon method (Dixon, 1984). Synthetic CT scans were generated from the in- and out-of-phase images using a commercial pretrained deep-learning algorithm (Figure 1; BoneMRI V1.6 research version, MRIguidance BV, Utrecht, Netherlands).

**TABLE 1:** Volunteer data.

Volunteer	Scanned levels	Deep-learning set IVD	Note
1	T4-L4	Test/train	
2	T1-L3	Test/train	
3	T2-L4	Test/train	
4	T4-L5	Test/train	
5	T6-S1	Test/train	
6	T3-L5	Test/train	
7	T3-L4	Validation	L4-S1 spine implant
8	T5-L5	Validation	Scoliosis with ∼30^o^ Cobb angle

### 2.2 Segmentation

**FIGURE 2:**
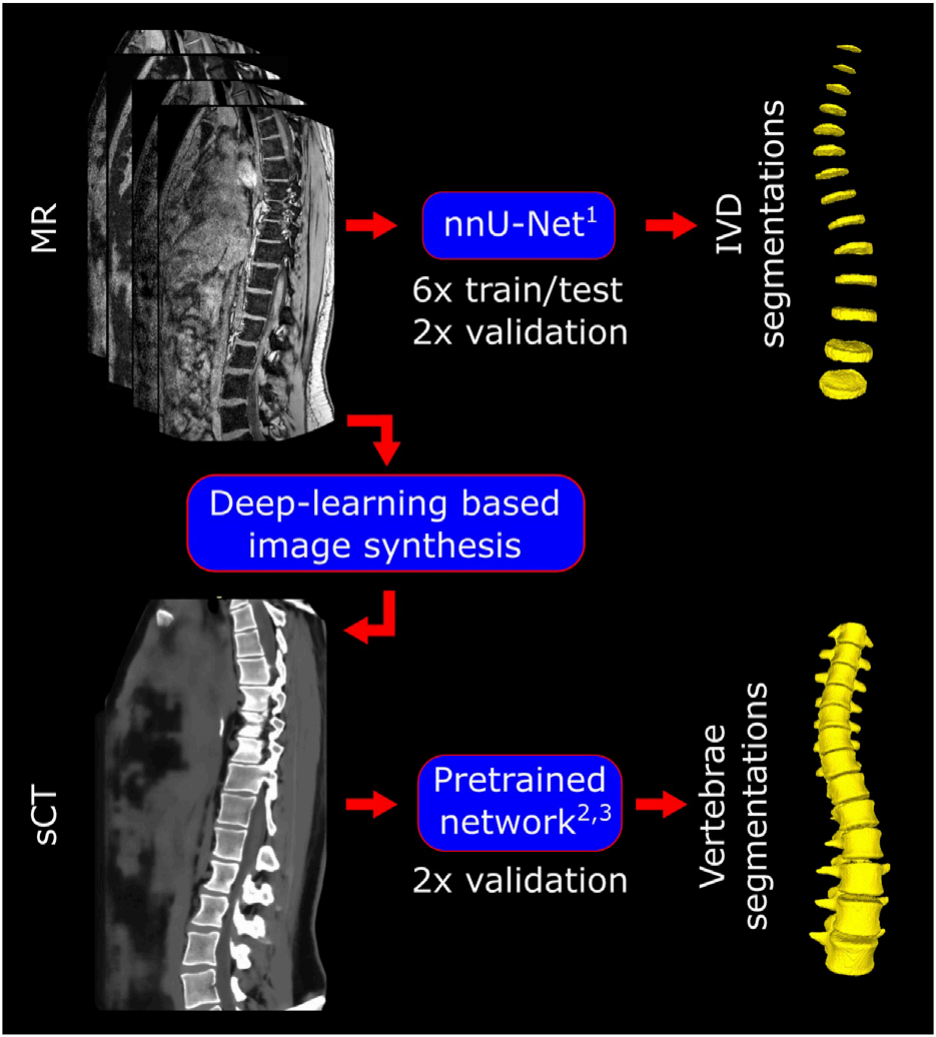
Pipeline for the manual and automatic segmentations. For the IVDs, the MR images were used and nnU-Net was trained, tested, and validated for the automatic segmentations. sCT images were synthesized by MRIguidance BV using their previously validated deep-learning based algorithm. An existing pretrained network was validated for the automatic segmentations of the vertebrae from these sCT images. ^1^(Isensee et al., 2021); ^2,3^(Payer et al., 2020; Sekuboyina et al., 2021).

An existing self-configuring deep-learning network (nnU-Net (Isensee et al., 2021)) was trained for the automatic segmentation of the IVDs (Figure 2). First the IVDs were manually segmented from the out-of-phase image stacks to represent the ground truth labels. The in-phase, out-of-phase, fat, water, and the ground truth label images of six healthy volunteers were then used for the training of the nnU-Net. This network includes training using five different test/train splits and automatic post-processing. Validation was performed on the remaining two volunteers, of which one with scoliosis and one with spinal implants.

Manual labelling and segmentation of the vertebrae was done on the sCT images of the volunteers with the implant and scoliosis. Automatic labelling and segmentation of the vertebrae was performed on the sCT images using a pretrained deep-learning network (Payer et al., 2020). This network has previously been trained and validated on the dataset of the Large Scale Vertebrae Segmentation Challenge 2020 (VerSe 2020 (Sekuboyina et al., 2021)). All vertebrae with more than half of their body inside of the field of view were included for validation between the automatic and manual segmentations.

For the validation, the accuracy of the automatic segmentations of all individual vertebrae and the IVDs in the validation sets is evaluated using two scores. First, the dice-score is calculated to quantify the overlap between the manual and automatic segmentation (Zou et al., 2004). A dice-score of 0 indicates no overlap between segmentations and 1 indicates perfect overlap. Second, the Hausdorff distance is calculated to find the maximum of all minimum distances between the automatic and the manual segmentations. This measure is bidirectional, measuring the distances from the automatic to the manual segmentations and vice-versa, and can thus capture both under- and over-segmentation. The region where the largest Hausdorff distance is calculated is the region where the automatic and manual segmentations differ most. For the volunteer with spinal implants the IVDs and vertebrae from T3-L2 were considered ‘healthy’ and unaffected by image artifacts coming from the implant.

### 2.3 Mesh morphing

**FIGURE 3:**
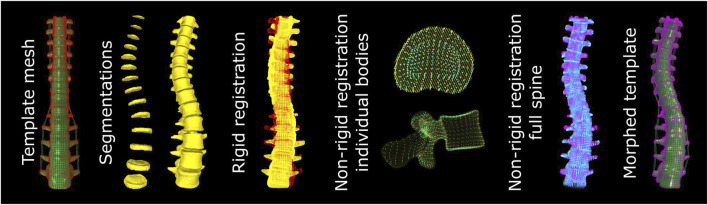
Pipeline for the morphing of the mesh. The template mesh is morphed in three steps. First, a rigid registration to the segmentations is applied. Second, the meshes of the individual IVDs and vertebrae are non-rigidly registered to the respective segmentations. Third, the complete mesh of the spine is registered to the non-rigidly registered meshes of the individual bodies to prevent a mismatch in alignment between individual bodies in the final mesh.

For the morphing of the spine a template mesh was extracted from a commercially available global human body model (GHBMC F05-P, Elemance, LLC, Winston Salem, United States). The vertebrae, IVDs, and ligaments from T1 to L5 were separated from the global model. The individual vertebrae and IVDs, and the section of the spine inside the field of view was then morphed to the segmentations using a previously developed, accelerated BCPD algorithm (Figure 3 (Hirose, 2021b; Hirose, 2021a);). Parameters that need to be defined were: Lambda, controlling the expected deformation vector length; Beta, controlling the range for the smoothing of the deformation vector; Omega, the outlier probability; Gamma, controlling the randomness of point matching at the beginning of the optimization; and c and n the convergence tolerance and maximum number of iterations, respectively. Some of these parameters were set to a fixed value and others were varied for different registration tasks (Table 2). The selected ranges for optimization were limited to improve the speed of the optimization. The upper limits were selected to lead to little deformation but high-quality meshes. The lower limits were selected to guarantee some self-intersecting elements, to ensure that the optimum would be between the limits. Default settings for acceleration based on the Nystrom method were used (Williams and Seeger, 2000).

**TABLE 2:** Parameter settings for the BCPD algorithm at the different steps. Where a range is given, the value was optimized within these limits.

	Rigid registration	Individual vertebrae	Individual IVDs	Complete spine
Lambda	1e9	10–2,500	1–4,000	0.1–50
Beta	2	0.25–1.5	0.5–2	0.5–2
Omega	0	0.01	0.01	0.01
Gamma	10	0.5	0.1	0.5
c	1e-6	1e-6	1e-6	1e-6
n	500	500	500	500

From the template mesh, point clouds were created of the nodes at the surface of each selected vertebra and IVD. Shared nodes between the IVDs and vertebrae were included in these surface node sets. From the segmentations, point clouds were generated based on the voxels at the surface of each vertebra and IVD in the field of view. First, the point cloud of the full spine section from the template was rigidly registered to the combined point clouds of the segmentations of the IVDs and vertebrae. Second, individual vertebrae and IVDs were non-rigidly registered. For this step the parameters used for registration were optimized for each body. In each iteration the registration accuracy was assessed by calculating the mean and maximum distance between the point clouds. Additionally, the mesh was rebuilt by linear interpolation of the deformation of the surface nodes. This mesh was checked for self-intersecting elements. The parameter Lambda was decreased each iteration, generally improving the registration accuracy until any self-intersecting elements were present. At that point the setting Beta was increased (increasing the smoothing of the deformation vector). Once the mean (for IVDs) or maximum (for vertebrae) distance between the point clouds no longer improved, the best Lambda of the second to last tested Beta were used for the final registration of the body. Third, the rigidly registered point cloud of the full spine section in the first step was registered to the collection of the individually registered bodies. This step was performed to ensure that the nodal connection between all vertebrae and IVDs is maintained. Optimization of the settings for the registration of the spine section was performed the same way as for the individual bodies.

The accuracy of the complete morphing cycle is quantified by measuring the distance from the surface nodes to the closest point on the surface of the segmentations. Additionally, the mesh quality of the morphed mesh is compared to that of the template. To define the mesh quality the Jacobian, maximum warpage, maximum aspect ratio, and maximum skew angle for each element are calculated.

## 3 Results

### 3.1 Segmentation

**FIGURE 4:**
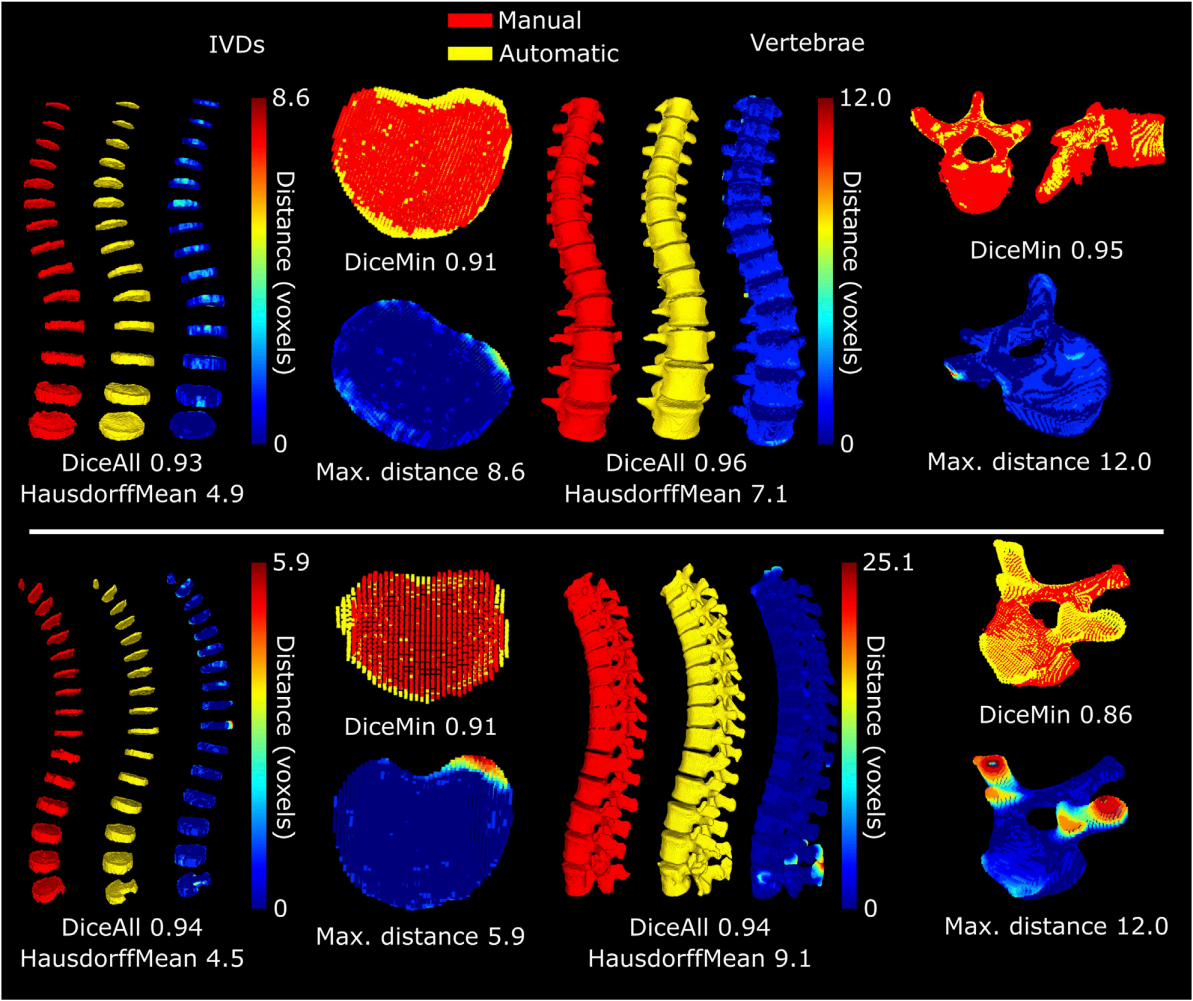
Accuracy of automatic segmentation for the subject with scoliosis (top) and the subject with implants (bottom). The selected IVDs and vertebrae for each subject are those where the dice-score was the lowest (top) or where the maximum distance from the automatic to the manual segmentation was the highest (bottom), excluding the region with the implant. The HausdorffMean refers to the mean of the maximum Hausdorff distances of the individual bodies.

**FIGURE 5:**
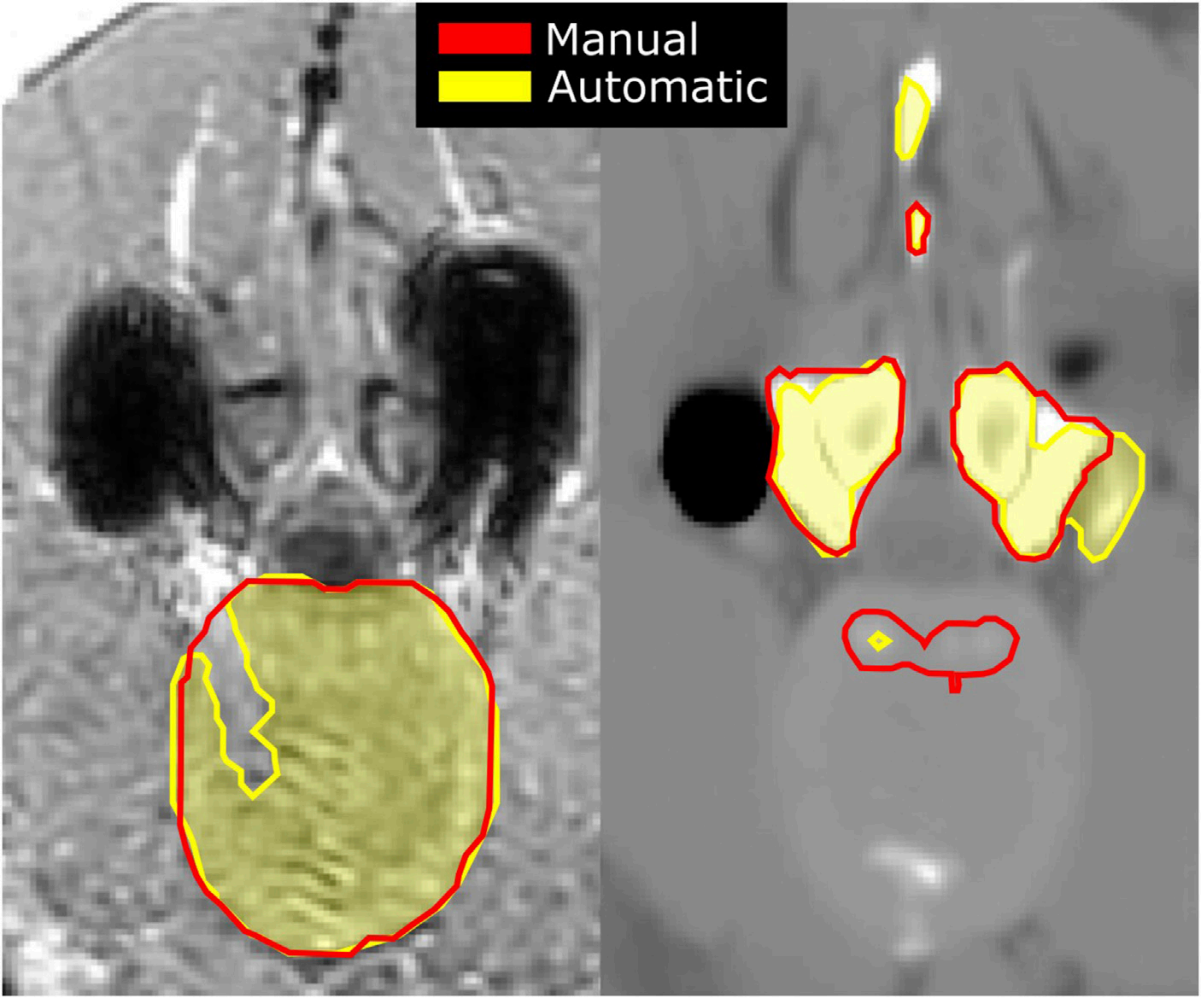
Artifacts originating from the spine implant on an acquired in-phase MR image (left), shown as dark signal voids, with manual (red) and automatic (shaded yellow) segmentations of the IVD; and an sCT image (right) of the same slice with artifacts originating from the signal voids leading to inaccurate segmentations of the vertebrae.

For the scoliotic spine, overall dice-scores of 0.93 (0.91–0.95) and 0.96 (0.95–0.97) were reached for the IVDs and the vertebrae, respectively (Figure 4). The largest differences between the segmentations of the vertebrae can be seen where the transverse processes reach outside the field of view. At these points the automatic segmentations do not consistently predict the bone to reach to the edge of the field of view, leading to under-segmentation. For the volunteer with implants, the achieved dice-score was 0.95 for all IVDs, excluding the L4-L5 IVD (0.84–0.97 for the individual IVDs). For the T3 to L2 vertebrae the dice-score was 0.95 (0.86–0.97 for the individual vertebrae). The lowest dice-scores come from the top of the spine (T3 and the T2-T3 IVD) because these are close to the top of the field of view where the image quality is lower and manual segmentations may be incomplete. Generally, for the rest of the IVDs the largest differences between the automatic and manual segmentations were found in the anterior and posterior regions. Here the automatic segmentations may include parts of ligaments or cartilage on the ribs. Due to the implant, artifacts were visible in the acquired MR images, and as result also in the sCT images which were reconstructed from the acquired MR images. These artifacts are inherent to the fundamentals of MR imaging. In this analysis, these artifacts led to a clear under-segmentation of the IVD near the pedicle screws (Figure 5). In the vertebrae this led to over-segmentation of the posterior processes, including at the vertebra above the level of the implant.

### 3.2 Mesh morphing

**FIGURE 6:**
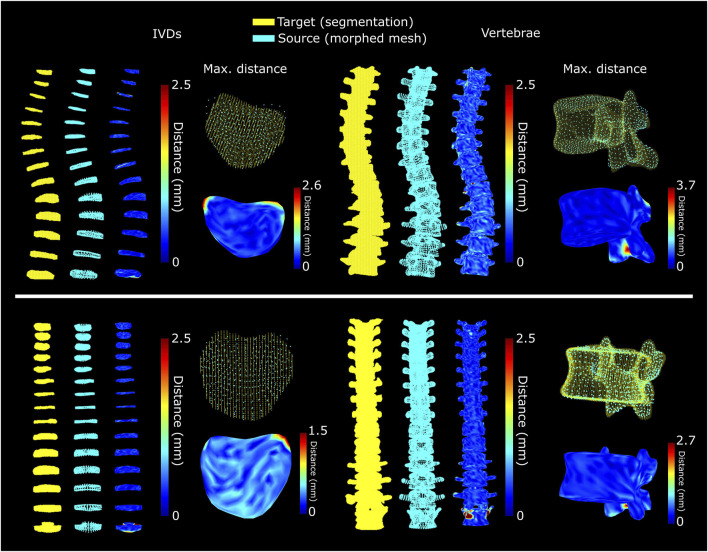
Separately optimized morphing of the discs and vertebrae for the subject with scoliosis (top) and for the subject with spine implants (bottom). The selected IVDs and vertebrae are those with the highest maximum distance between morphed mesh and segmentation, excluding the region with the implant.

**FIGURE 7:**
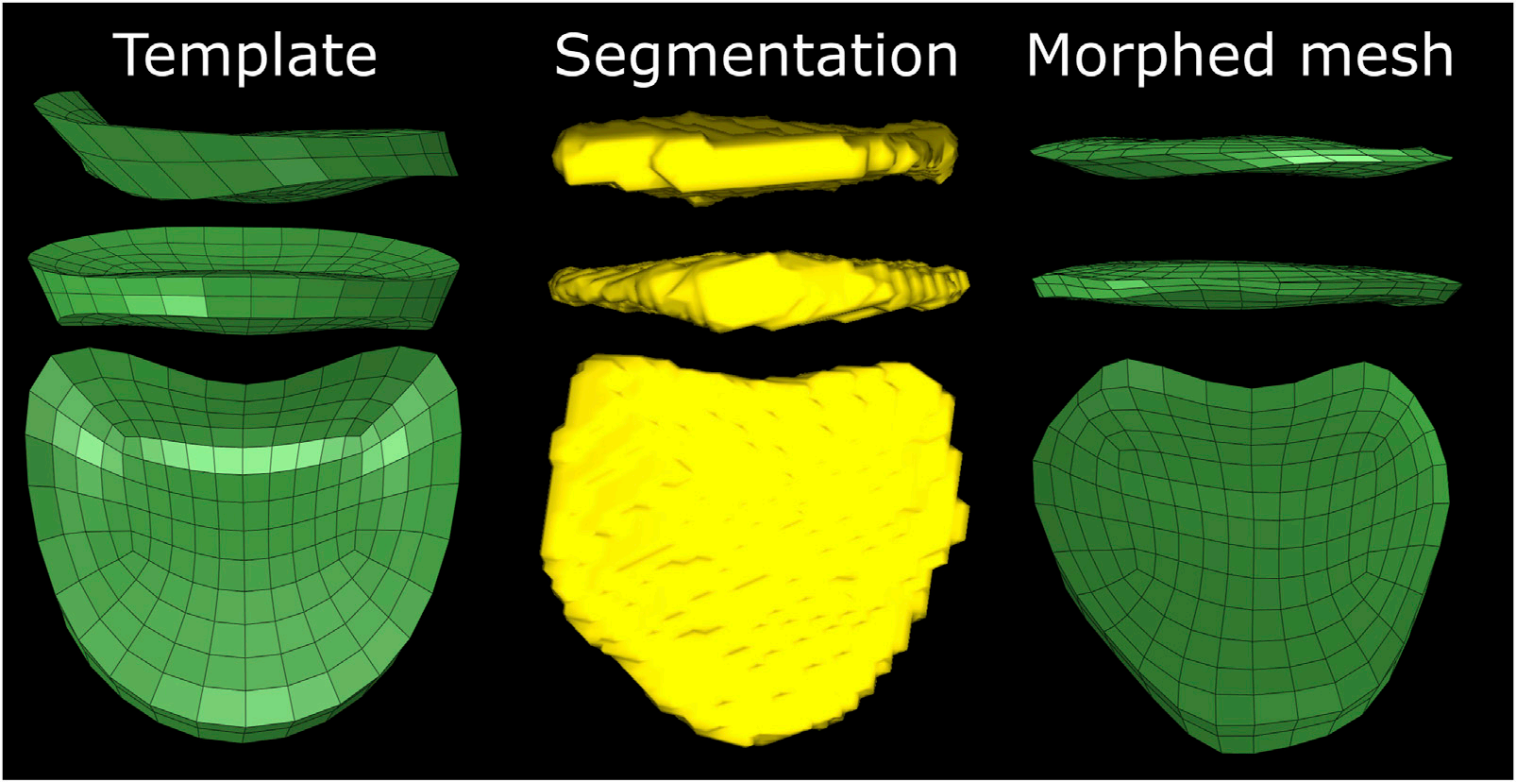
Initial difference between template mesh, automatic segmentation and the morphed mesh of the T6-T7 IVD of the subject with implants. The irregular shape and presence of the narrow region at the posterior side of the template leads to skewed elements.

For the scoliotic spine, the mean (SD) distance for all of the individually morphed IVDs to the automatic segmentations was 0.39 (0.16) mm (Figure 6). The maximum distances ranged from 0.7 to 2.6 mm for the individual IVDs. The highest distance was at the IVD between T6 and T7, where high deformations are required due to the large initial difference between the template IVD and the subject-specific IVD (Figure 7). The distance for all vertebrae was 0.46 (0.24) mm. The maximum distances varied from 1.3 to 3.7 mm for the individual vertebrae, with the maximum distance at T12 between the facet joints. For the volunteer with implants, the mean distance from the individually morphed IVDs, except the L4-L5 IVD, to the automatic segmentations was comparable to that of the scoliotic spine (0.39 (0.14) mm). For the vertebrae, the mean distance for the individual T3-L2 vertebrae (levels superior to the implant) was 0.42 (0.18) mm.

**FIGURE 8:**
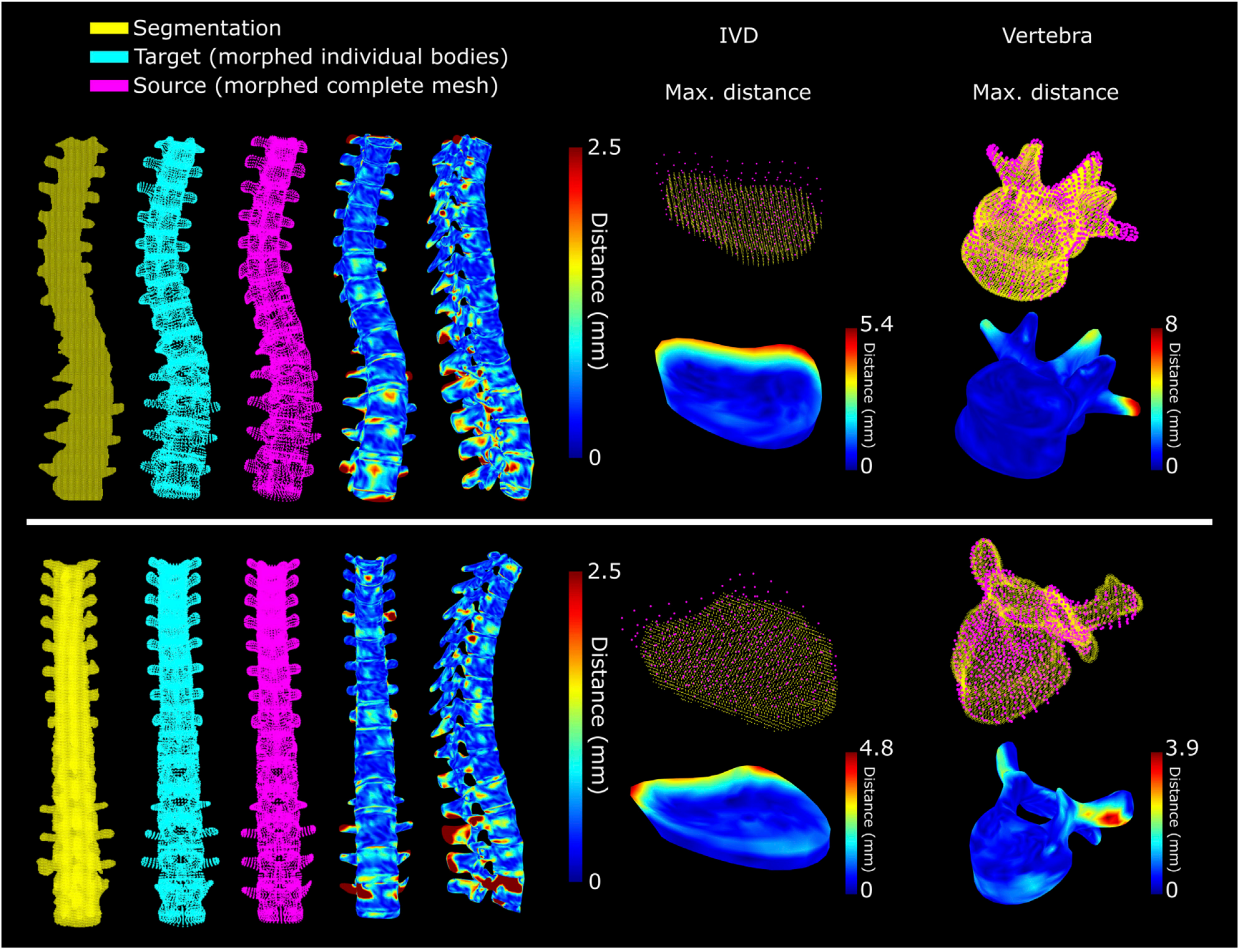
Accuracy of mesh morphing of the complete spine for a subject with scoliosis (top) and for a subject with spine implants (bottom). The selected IVDs and vertebrae are those with the highest maximum distance between morphed mesh and segmentation, excluding the region with the implant.

To ensure a good connection between the elements of the IVDs and vertebrae, the complete spinal segment was morphed to the individually morphed bodies. When morphing the complete spine, the mesh remains more similar to the template compared to when the individual bodies are morphed. For the volunteer with scoliosis, morphing of the complete spine led to a mean (SD) distance of 0.73 (0.54) mm for the IVDs with maximum distances ranging from 1.6 to 5.4 mm for the individual IVDs (Figure 8). For the vertebrae, the mean distance was 0.78 (0.70) mm with the maximum distances ranging from 2.9 to 8.0 mm for the individual vertebrae. For the volunteer with implants, morphing of the complete spine led to a mean distance of 0.74 (0.64) mm for the IVDs with maximum distances ranging from 1.6 to 8.2 mm for the individual IVDs (Figure 8). For the vertebrae, the mean distance was 0.77 (0.90) mm with the maximum distances ranging from 2.1 to 15.8 mm for the individual vertebrae. The largest distances can be seen at the posterior processes at the height of the implant. The maximum distance for T3-L1 was 5.2 mm.

**FIGURE 9:**
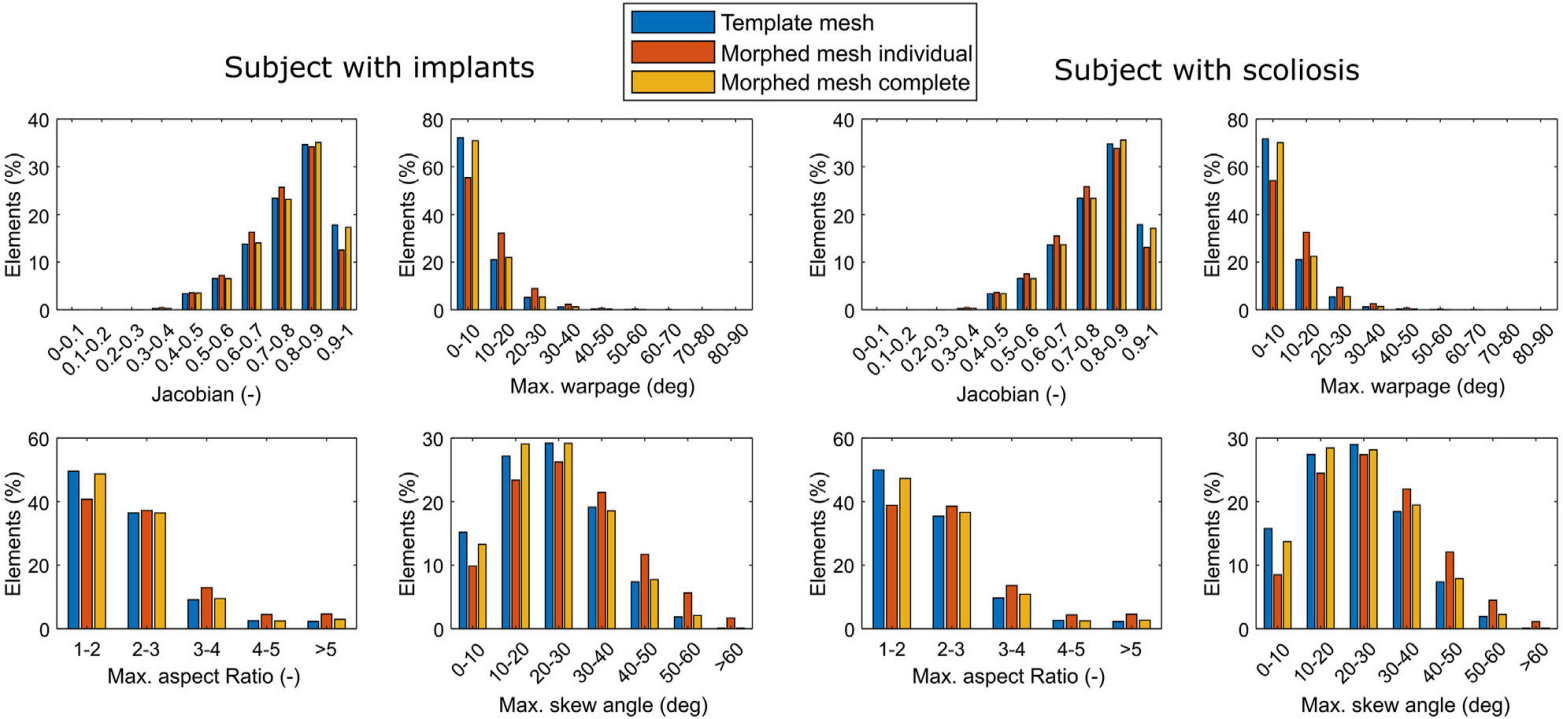
Shape quality of the elements of the template, individually morphed, and completely morphed mesh of the subject with scoliosis.

The quality of the final mesh was similar to that of the template mesh for both subjects (Figure 9). In both cases overall mesh quality was high, with more than 96% of the elements having a Jacobian >0.5, a maximum warpage <30°, a maximum aspect ratio <5, and a maximum skew angle <50°. The morphing of the individual IVDs and vertebrae led to a larger reduction of the mesh quality. The lowest mesh quality was found in the T6-T7 IVD of the subject with implants, where 64% of elements had a maximum skew angle >50° (Figure 7).

## 4 Discussion

In this paper a new approach for the automatic generation of FE models from MR and synthetic CT images is explored. It was found that using two existing deep-learning networks the IVDs and vertebrae can be accurately segmented from MR and sCT, respectively. It was also found that using a BCPD algorithm a template mesh can be registered to the automatic segmentations resulting in a high-quality subject-specific mesh that can be used for FE modelling. The images acquired in this study cover a large field of view, including the lumbar and thoracic spine. The use of sCT images enabled the segmentation of bone and IVD from 1 scan that takes approximately 5 min scanning time. In this way, subjects are not exposed to harmful radiation and the images are inherently aligned alleviating the need for co-registration. The template mesh was extracted from a commercially licensed model of a 24-year old female, where the skeletal structures are based on CT images. This template was selected because it is an accurate geometrical representation of a healthy spine. Tests with other template models (results not shown), such as the openly available Total Human Model for Safety (Toyota Motor Corporation, Aichi, Japan), provided similar results, indicating the feasibility of using the morphing method with other templates.

The automatic segmentations work well with high dice-scores for the complete spine and for the individual IVDs and vertebrae. The network used for the segmentations of the vertebrae was pretrained on an existing set of 374 CT images that were made available for the Large Scale Vertebrae Segmentation Challenge 2020 (VerSe 2020 (Sekuboyina et al., 2021)). The validation performed on the images in that set resulted in dice-scores of 0.91, lower than the 0.94 and 0.96 from validation against the subjects in this study. This is an indication that the sCT images are well-suited for the automatic segmentation of vertebrae. The network used for the segmentation of the IVDs was not previously trained on IVDs but also performed well. Dice-scores were higher than achieved for a challenge posed in 2018 (Zheng et al., 2019), where the best models entered reached a dice-score of 0.91, and another trained on the same dataset (not entered in the challenge) reached 0.92 (Dolz et al., 2018).

The largest differences between the automatic and manual segmentations of the vertebrae were found near the edges of the field of view and near the spine implant. In these regions the sCT images are dissimilar from true CT images. Even though the training set for the network used for the segmentation of the vertebrae included spines with implants, the dissimilarity between the artifacts in true CT and sCT is too large. Accurate segmentation in these regions on sCT is currently impossible due to the nature of MR. With MR no signal is obtained around metal implants, which leads to fundamentally different artifacts on sCT compared to metal artifacts in true CT images. In the IVDs the highest inaccuracies were found in their anterior and posterior regions. These regions coincide with the location of the posterior and anterior longitudinal ligaments. The ligaments are mostly indistinguishable from the annulus fibrosus on the MR images, which also led to inconsistencies in the ground truth manual segmentations.

Morphing using the publicly available BCPD algorithm (Hirose, 2021a) works well, especially for spines where individual bodies (vertebrae and IVDs) have a near-normal shape. In the case of uncommon features, such as artifacts due to implants, the morphing of the complete spine still leads to a high-quality mesh, but slightly deviates from the segmentation. While in this case this is due to image artifacts, it is possible that pathologies that can results in large variations in local shape (e.g., lesions; Schmorl nodes; herniated IVDs) will also not be meshed accurately. However, due to the multi-scale nature of the presented method, these local variations can be captured when morphing individual bodies. The optimization ensures that the resulting mesh can be used for FE modelling. However, an initial guess of set parameters and sufficiently large discrete steps are required. An exhaustive parameter sensitivity study and improvement of the optimization algorithm is expected to lead to some further improvement. In the current implementation, the template mesh has a clear influence on the outcome when morphing the complete spine. An adaptation to the algorithm could be developed to connect the individually morphed bodies maintaining the accuracy of the local morphing. Alternatively, a multi-block approach could be implemented. This has been proven to give good results for the generation of meshes for thoracic spines with Cobb angles up to 90° (Hadagali et al., 2018). A disadvantage of such a technique would be that re-meshing of individual blocks is required, thus not maintaining the element connections in the template.

A limitation of this study is that not all biological variation that is relevant for the biomechanics of the spine are included in the presented segmentation and morphing strategy. The annulus fibrosus and the nucleus pulposus are morphed as one, even though changes in size and location of the nucleus can lead to significant changes in, e.g., predicted peak force under compression (Du et al., 2021; Liu and El-Rich, 2020). A T2-weighted MR sequence could be added for the separate segmentation of the nucleus pulposus (Castro et al., 2014) so that it can also be included in the morphing. Another limitation is that the creation of the sCT images has not yet been explicitly validated for the thoracic spine. However, clinical evidence is already available for the hip joint (Florkow et al., 2022), and the lumbar (Morbée et al., 2021; Davidar et al., 2023) and cervical spine (van der Kolk et al., 2022). These studies extensively validated the similarity between segmentations from sCT and true CT, indicating that the accuracy of the sCT images is more than sufficient for the segmentation of the vertebrae. Additionally, the Hounsfield units in the sCT images are similar to that of true CT images (Florkow et al., 2020; van der Kolk et al., 2022). Accurate measurements of the Hounsfield units means that these images can also be used to derive the bone density and map material properties such as the Young’s modulus to elements in the FE models (Taddei et al., 2007). A lesser limitation is that the facet joints are not explicitly included. Finite element models of the spine have shown good results without explicitly including a cartilage layer and, for example, using a soft contact definition between vertebrae (Mengoni, 2021). However, the 3D shape of the joint is important for a proper understanding of its function (Inoue et al., 2020). Separate segmentation and modelling of the facet joints from the MR images could lead to improved biomechanical models of the spine.

## 5 Conclusion

The new approach presented in this study can be used for the automatic generation of subject-specific spine models. It can be used for both the morphing of individual vertebrae and IVDs and for the morphing of a complete spine. This allows for the creation of multi-scale FE models with little effort. Additionally, the automatic segmentations could independently be used for morphometric measurements of the IVDs and vertebrae. For improved subject-specific biomechanical measurements, segmentation and morphing of the nucleus pulposus and facet joints should be added.

## Data Availability

The datasets presented in this article are not readily available because the clinical data used in this study cannot be made available to third parties. Requests to access the datasets should be directed to BR, b.v.rietbergen@tue.nl.
